# Transforming Alcohol and Other Drug Education Through Co‐Design

**DOI:** 10.1111/inm.70009

**Published:** 2025-02-10

**Authors:** Mark Goodhew, Sonia Matiuk, Carmen Axisa, Chris Gough, Jo River

**Affiliations:** ^1^ School of Nursing and Midwifery Western Sydney University Sydney New South Wales Australia; ^2^ Faculty of Health University of Technology Sydney New South Wales Australia; ^3^ Canberra Alliance of Harm Minimisation & Advocacy Canberra Australia

**Keywords:** co‐design, consumer participation, evaluation research, health education, qualitative, substance use

## Abstract

Over the past decade, as a broader push to address community needs and improve health outcomes, socially marginalised people have become increasingly involved in co‐design research and education. More recently, people with lived experience of substance dependence have co‐designed and co‐delivered Alcohol and Other Drugs (AOD) education with nurse educators. Evaluations of co‐designed AOD education have largely focused on educational and clinical outcomes but not on the process of co‐design. Because of this gap, there is a need for research that explores the process of undertaking co‐design with people who use substances and clinicians, including facilitators and barriers to the process that are specific to these groups. This research reports on a qualitative study exploring the barriers, facilitators, and perceived value of a co‐design process for an AOD clinical education program involving people with lived experience of substance use, nurse clinicians, and academics (nursing and public health). The participants reported that building trusting relationships and skilled facilitation enhanced the co‐design process, enabling positive conditions for working in partnership to achieve the project outcomes. However, challenging interpersonal dynamics, lack of adequate resources, and overly directive facilitation could impact team dynamics and undermine the co‐design process. Authentic co‐design has the potential to transform AOD education, ensuring that it centres the needs of people with lived experience. However, co‐design initiatives require adequate resourcing and time to develop equal and respectful relationships for this vision to be realised.

## 1 Introduction

Over the past few decades, internationally, frameworks and policies have emerged to involve people with a lived experience of mental health challenges and/or substance (alcohol and other drugs [AOD]) use in research (River et al. 2023; National Health and Medical Research Council 2018); service delivery (Roper, Grey, and Cadogan 2018; Goodhew, Stein‐Parbury, and Dawson 2018); and education (Arblaster et al. 2023; Horgan et al. 2018). Involvement of people with lived experience can increase the relevance of research, education and service priorities, enhance the quality of knowledge implementation and translation (Brett et al. 2012), and is predicted to become the “new normal” in research‐informed policy, education and services (Jones 2022, p15). In this article, people with lived experience refers to those with lived and/or current living experience of substance use.

Co‐design is an approach to educational design that aims for genuine partnership and involves equal numbers of people with lived experience who co‐decide with other key stakeholders, including clinicians and academics, co‐deciding through the planning, design, delivery, and evaluation stages of an educational initiative (Brand et al. 2021). While there is an increasing move towards co‐designed education with people with lived experience of mental health challenges (Happell, Platania‐Phung, et al. 2019; Happell, Waks, et al. 2019; Horgan et al. 2018), people with lived and living experience of substance use are rarely involved (Moll et al. 2020). It is only more recently that people with lived experience of substance use have co‐designed and co‐delivered AOD undergraduate nursing education, with findings indicating that it reduced students' stigmatising views and was perceived as improving their skills to collaborate with people who use AOD (Goodhew et al. 2023, 2021). Despite indications of interpersonal and structural barriers to meaningful participation of people with lived experience in collaborative endeavours (Patterson, Trite, and Weaver 2014), little has been reported on the barriers to participation for people with lived experience of substance use and factors that enhance the co‐design process.

This article reports on the co‐design process of AOD education for health professionals in clinical practice undertaken by a team of nursing and public health academics, clinicians, and people with lived experience of substance use who have expertise in AOD advocacy and peer work.

## 2 Background

### 2.1 Towards Co‐Design of AOD Education

AOD use is a public health issue in Australia and worldwide. Globally, 2% of people are diagnosed with substance dependence, and AOD use is predicted to increase by 11% by 2030 (UNODC 2021). There are 11.8 million AOD‐related deaths worldwide (Ritchie 2019). Australia reflects global trends, with one in four people exceeding guidelines for safe alcohol use and 43% of Australians aged 14 overusing non‐prescription drugs in their lifetime (Australian Institute of Health and Welfare [AIHW] 2022b), 1559 alcohol‐induced deaths in 2021 (AIHW 2022a) and 1842 drug‐related deaths in 2020 (AIHW 2022b).

Clinicians who can intervene effectively are vital in reducing mortality and morbidity associated with AOD use (Tierney et al. 2020). As the largest healthcare discipline, nurses are often the first and sometimes only contact for people with substance‐related health concerns (Tierney et al. 2020). However, clinicians often receive little training in AOD use and lack the confidence and skills to provide adequate care (Compton and Blacher 2020; Ford 2011). Research also suggests that clinicians often hold stigmatising views towards AOD patients, including that they are manipulative, drug‐seeking, and undeserving of care (Copeland 2020; Horner et al. 2019; Neville and Roan 2014). Early life traumas are associated with emotional dysregulation and substance use (Barahmand, Khazaee, and Hashjin 2016), but clinicians often misjudge consumers' distress as ‘bad behaviour’ (NADA 2022). This can lead to discriminatory behaviours that can impact access to care for people who use substances, who may avoid seeking and engaging in health services (Simon, Snow, and Wakeman 2020, van Boekel et al. 2013). Involving people with lived experience of substance use in the co‐design and co‐delivery of AOD education has the potential to centre lived experience perspectives, shift healthcare clinicians' stigmatising views and enhance their ability to support people who use substances (Goodhew et al. 2023).

Involvement of people with lived experience occurs on a spectrum from non‐participation (e.g., excluded, informed, or subjects in research or education), to consultation (can voice issues but have no influence), to genuine partnership or leadership in education, research or service design (River et al. 2023). Increasingly, there has been a push for genuine partnership education and research, including co‐design approaches. Co‐design is an approach that aims for equitable engagement with those most impacted and ideally involves equal numbers of people with lived experience who work alongside other key interest‐holders (e.g., clinicians and researchers), co‐deciding through all stages of planning, design, delivery, and evaluation, and dissemination of an initiative (Bellingham et al. 2023). Attention to co‐design has arguably stemmed from calls from consumer and peer movements for parity in design and evaluation processes (River et al. 2023) and recognition of human rights violations against people with lived experience (Campbell and Rose 2010).

People with lived experience of social marginalisation (i.e., people with lived experience of mental distress, homelessness, substance use, etc.) are increasingly involved in co‐designing research, education, and service design (Happell, Platania‐Phung, et al. 2019; Happell, Waks, et al. 2019; Horgan et al. 2018). Co‐designed education offers tremendous potential to galvanise educational and community benefits. For example, people experiencing homelessness have partnered in the co‐design of services for homeless populations, and people with lived experience of mental distress have been involved in the design and delivery of clinical education (Brand et al. 2021; Mullins et al. 2021; River et al. 2023). This has provided important opportunities for lived experience communities to contribute to clinical education and change health professional practice (Bellingham et al. 2021; Brand et al. 2021; Mullins et al. 2021; River et al. 2023). However, co‐designed clinical education research has mostly focused on outcomes, and few studies have concentrated on the co‐design process. The small body of research on co‐designed education reports that factors such as trust, respect, inclusiveness, rapport, common goals, and hospitality unite the co‐design team and facilitate the process, while academic bureaucracies and unequal power among team members can hinder co‐design initiatives (Brand et al. 2021; Molloy et al. 2024).

Co‐designed and co‐delivered educational initiatives are rare in the context of AOD use (Goodhew et al. 2023; Roussy et al. 2015; Valenti and Allred 2020). Between 2019 and 2021, Goodhew et al. (2021) co‐designed, co‐delivered and co‐evaluated tertiary‐level AOD undergraduate nursing curricula that embedded lived experience perspectives. The evaluation findings indicate that the program improved student nurses' perceived knowledge and skills in evidence‐based, collaborative and trauma‐informed AOD care and reduced stigmatising attitudes towards people who use substances (Goodhew et al. 2023). Extending this work, in 2022, our team co‐designed AOD education for healthcare workers in clinical practice, which is currently being implemented and evaluated in health services. However, despite considerable barriers to collaboration in co‐design, including stigma, discrimination, inflexible timelines and bureaucracy (Molloy et al. 2024; Patterson, Trite, and Weaver 2014), little has been mentioned in the research about the specific barriers to successful co‐design of AOD education and factors that support collaboration. Research on the co‐design process with people with lived experience, clinicians and academics has the potential to inform educators seeking to partner with people with lived experience of substance use genuinely.

This article seeks to address this gap and reports on an investigation of the experience of co‐designing AOD education, including barriers, facilitators, and perceived value of the co‐design process.

## 3 Methods

### 3.1 Methodology

A qualitative descriptive methodology was used to evaluate the co‐design process. The qualitative descriptive method has become popular in nursing education, as it is useful for studying novel phenomena and enables the researchers to stay close to participants' meaning of an event (Sandelowski 2010).

### 3.2 Co‐Design of the AOD Education

Between May and August 2022, five people with lived experience, three clinicians (registered nurses specialising in AOD) and three academics (nursing and public health) came together to co‐design an AOD educational program for clinicians and peer workers who work in mental health and AOD services. Table 1 outlines the three co‐design modules of the educational program, which will occur via a study day and co‐delivered by people with lived experience, clinicians, and academics. The co‐design meetings were facilitated by an experienced co‐design facilitator (MG), who used co‐design principles and practices to ensure equitable dialogue and decision‐making among team members, including people with lived experience, clinicians, and academics.

**TABLE 1:** Co‐designed AOD education program's modules.

Module 1	Module 2	Module 3
AOD and service use from a lived experience perspective, including: The origins and impacts of stigma and discrimination Inclusive language for AOD service provision The relationship between AOD use, complex trauma, and the social determinants of health	Best‐practice approaches to the care of people who use AOD, which include: Building a therapeutic alliance with people who use AOD Trauma‐informed care and practice Harm reduction principles and practice	Collaborative, therapeutic and person‐centred AOD assessments and care planning, including: Strengths‐based care planning Goal setting, prioritisation and review for Clinical skills that promote help‐seeking and harm‐reduction

### 3.3 Data Collection and Analysis

Following approval from the Human Research Ethics Committee, co‐design team members were invited to participate in semi‐structured interviews. A semi‐structured interview topic guide was prepared by MG and JR, and was based on the aims of the research, and informed by extant literature on co‐design processes in health research. Four people with lived experience and four nurses (two clinicians and two academics) consented to be interviewed. The interviews lasted 14–44 min (average 33 min) and were digitally recorded and transcribed verbatim. The data were de‐identified before analysis. Data analysis was conducted using Braun and Clarke (2022) six‐stage reflective thematic analysis, including: data familiarisation, coding, development of categories and themes (MG, JR, CA, SM), and review of themes and write up (MG, JR, CG).

## 4 Results

Two main themes emerged from the analysis. The first theme, ‘creating the conditions for working together’, highlights necessary elements of collaborative working, and the second is ‘valuing the co‐design outcome’, which examines the motivation of people in the co‐design process as an important aspect of its success.

### 4.1 Creating the Conditions for Working Together

The data revealed that participants perceived effective co‐design as requiring teams to build trusting and equitable relationships, which was supported through skilled facilitation and adequate resources.

#### 4.1.1 Building Trusting and Equitable Relationships

The data indicate that trusting and equitable relationships were important for mutual exchange and learning in the co‐design process and that this often took time to build.

People with lived experience indicated that it took time to trust the co‐design process, and they were initially nervous and wary of nurses, particularly if they had negative experiences in health services. As one participant stated:

It's always a bit nerve‐wracking at the start when you don't know who the people are that you're in a room with, and what their attitudes are, or what their ideas are. Because, I think they're a minority, but I have met a few people who work in drug and alcohol who have some very old‐fashioned ideas around abstinence …, and they don't really know a lot about harm reduction. (Person with lived experience 3)

Despite being debriefed about the importance of fostering equitable relationships and using non‐stigmatising language, one participant stated, [the nurse said] Oh, they [people who use drugs] all act like children or something like that' (Person with lived experience 3). Statements of this kind by clinical team members had significant impacts on the co‐design process, with lived experience participants noting that it made it difficult to establish trust if nurses in the team were ‘bossy’ or used stigmatising language. Participants indicated that trust could be difficult to establish if nurses in the team were ‘bossy’ or used stigmatising language.

Despite these challenges, multiple factors appeared to support the development of trust in the co‐design team. As the following quote suggests, having equal numbers of people with lived experience as clinicians in the co‐design could mitigate against nurses dominating the co‐design process:

There were three consumers around the table [with the nurse] and as I said, it … worked okay but had there been one consumer, that might have actually deafened their voice. They all knew each other, so they had this strong added bond and backed each other. So I think that kind of—again … they were happy to speak up. (Nurse 3)

Participants with lived experience also reported that the willingness of nurses and academics to listen to their views supported them in expressing their perspectives. As one person with lived experience noted:

But we were made to feel very comfortable; we were listened to; questions were asked of us; we had information to impart to other people. It was quite good, yes. (Person with lived experience 1)

Nurses also indicated that attention to power dynamics could support co‐design teams to build trust. Some nurses noted that they were aware that people with lived experience might feel more wary to express perspectives, and they deliberately sought to create a ‘safe space’ (Nurse 2) for people with lived experience to speak and be heard. As one nurse explained:

It definitely also allowed me to see just how important it is for the spaces, for the people who don't usually get to speak to be able to speak, and the restraint that's required from people who are used to speaking. (Nurse 1)

Nonetheless, despite nurse participants' efforts to create a safe environment, participants with lived experience were initially more cautious, and it took time to trust that the environment was non‐judgemental and feel the necessary degree of safety to express perspectives in the co‐design team. Eventually, however, with consistent support from facilitators and nurse participants, people with lived experience began to feel sufficiently safe to express their perspectives:

Most of the peers [people with lived experience] felt like they could be open and say what they thought in front of the nurses present without being judged. (Person with lived experience 3)

In an environment of sufficient trust, people with lived experience, academic and clinical nurses, could engage in mutual learning and discussion and find a common goal, that is, improving education to improve care for people who use AOD services. A participant stated:

I learned, just little bits and pieces from their side of the fence… It really made me realise, I may know shitloads about being in addiction but as I proclaim myself, it's a multifaceted thing, and I know fuck‐all about, excuse me, the other facets. And it was so—good doesn't cover it enough, it was just wonderful meeting others and learning from them. (Person with lived experience 1)

As this quote indicates, trust between team members improved team mutuality and capacity to understand the perspectives of service providers. As such, lived experience participants noted that the co‐design process had the potential to enable them to develop an educational program that was relevant to clinicians while remaining true to the needs of people who use substances. Nurses also noted the potential for the co‐designed education to be a driver of change, and to improve treatment and care for people who use substances. As one participant stated:

People went there wanting to be an actual driver of change. People went there with not a personal agenda. People went there knowing that we had a really marginalised group that are treated judgementally in the health system quite often, and so we wanted to try and see if we could try and address many of the current issues around drug and alcohol dependence within health systems. (Nurse 4)

#### 4.1.2 Skilled Facilitation and Resources

Data indicated that participants found a welcoming atmosphere and skilful facilitation of co‐design sessions, which was vital for mitigating the impacts of power differentials and ensuring the success of the process.

From the outset, the facilitator supported access, including allowing people with lived experience to bring dogs. As a participant with lived experience noted, this was ‘a very brilliant thing’ as they ‘didn't have to leave my dog at home.’ (Person with lived experience 4). Creating a welcoming atmosphere was perceived as particularly important to all participants:

I really liked about it is we were made to feel very welcome, given there's a lot of stigma associated with lived experience of drug use. (Person with lived experience 1)

Participants noted that the facilitator enhanced connection and respect through a careful introduction process:

…how he had met them. Putting the person in the context so we could see a more richer sense of the person but also him and the person, “This is how we met,” which I found really quite novel. I hadn't come across that before. (Person with lived experience 1)

Also vital was ensuring that all participants were included in discussions. As one participant said, ‘I felt that [the facilitator] has this particular skill where he can involve everybody’ (Person with lived experience 2). The participants also indicated the importance of how the facilitator consolidated ideas expressed in the co‐design meetings into coherent educational modules:

I was seeing was quite a lot of genius in it. … Putting together all this information into—not only bullet points, but into topics and a path for it to go forward. I thought was really good. It really blew me away. I thought he did that extremely well. (Person with lived experience 3)

However, in addition to the support of a facilitator, one lived experience participant, who had expertise in advocacy and peer work, took it upon himself to provide additional supports to lived experience members, taking responsibility for ensuring and supporting the participation:

I was the coordinator of all the people with lived experience, and it can be like herding cats sometimes, but everyone turned up on time, and if they were running late, they let us know, which was just a polite thing to do. (Person with lived experience 2)

Additionally, hospitality measures, including the provision of refreshments, also enhanced relationships and connections that supported the co‐design process:

It was good having the food at the start and having just a general chat and finding out where people were from. We actually spent a good 20 minutes, half an hour, just talking before we actually started, so I feel like I met people informally before we started so I think that was really good. (Person with lived experience 3)

Nonetheless, participants discussed points of improvement regarding facilitation, including a need to ensure that all members of the co‐design were comfortable with having a dog in the room. As one nurse noted:

One of the people with lived experience brought their dog …, and that was good … Obviously, it was a comfort dog, and—But it probably would have been reasonable just to double‐check with everybody that they were also comfortable … to agree for the doggie to be there, or to acknowledge that—is everybody uncomfortable, or not. (Nurse 1)

One participant also indicated that facilitation could sometimes be experienced as overly influential. While co‐design involves all members as active participants in the co‐design process, the facilitator's perspective could bear more weight and influence in the process:

I know there's a very fine line—a very delicate balance between keeping things moving and having structure and process, but allowing enough time for things to happen. Sometimes, [the facilitator] did lead more than I expected around things like telling us how he thought the training could be structured, or suggesting how it might be structured. And of course, because he's in that lead role, as soon as he makes that suggestion, it's giving a sense that that's what's expected that we'll do (Nurse1).

Interestingly, this participant notes that having more time to ‘let things happen’ and less of an agenda may make the process more equal. Nonetheless, another lived experience participant expressed frustration at peers who ‘rambled’ and ‘hogged the floor’ when there was so little time, although they acknowledged that this ‘happens in every group, and it wasn't a big deal’ (Person with lived experience 1).

Most participants mentioned that a lack of resources for co‐design could undermine the process. For example, restricted funds impacted time frames, and some participants with lived experience felt there was not enough time to express their ideas fully, which created a sense of uncertainty:

But we just don't know where we're going for the future of it. It would be good to have 12 weeks of program rather than, we go in for five weeks and there's another funding thing, and then there's another—it would be great to know we're doing this, to this point, and we move on. (Person with lived experience 4)

Lack of funds could also lead to a sense of sadness and relational conflict. As one participant with lived experience stated, ‘It became such a very good group dynamic, it was—I don't know—a degree of sadness to see it finish’ (Person with lived experience 3).

Findings from the creating conditions for working together theme indicate that effective co‐design requires relationships founded on trust and equity, skilled facilitation, hospitality, and adequate resources and time to allow everyone in the team to contribute to the process effectively.

### 4.2 Valuing the Co‐Designed Outcome

Another key theme related to the perceived value of the co‐designed educational program. Participants noted that the potential value lay in the synergy of lived experience and clinical and academic perspectives as it provided a more accurate, richer, and humanistic portrayal of substance use that could enable nurses and clinicians to reflect on their attitudes towards people who use substances. Indeed, as one participant noted, without these diverse perspectives, educational offerings are potentially ineffective:

The word that came to mind is paucity, like a poverty of, it would be impoverished, that's the word I'm looking for. The final product, either from experts or clinicians, without the other, it would be impoverished. Dare I say it would also be inaccurate in some regards, and therefore ultimately ineffective, not as effective as when it's brought by the two of us, like the two—yes, that's it. (Person with lived experience 1)

Participants also noted the importance of co‐delivery of co‐designed education:

I think it's innovative. I think to have the affected population to not just codesign but co‐deliver the education is much more powerful. It's evidence based by real stories and real people. … So I think it's really important for nurses to understand all of the behaviour and the physiological impact of alcohol and other drugs, but in a meaningful way. It's not just textbook. Anyone can read a textbook. This is just—it's almost like taking the stories and translating it. (Nurse 3)

I think it'll [co‐delivered education] help to confront people's personal stigmas, confront people's misconceptions about people who use drugs. It shows that, even though a person might have used drugs for quite a number of years, they're not running around with two heads; they're not all what you see in the Daily Telegraph. (Person with lived experience 2)

The participants believed that being taught by people with lived experience would help clinicians see beyond a person's stigmatised identity, enabling them to better connect, collaborate, and care for people who use AOD. Ultimately, participants held the view that co‐designed education could lead to better clinical care, improved communication between clinicians and service users, and more job satisfaction for clinicians:

It will certainly bring value, because one of the main problems of drug users being admitted to hospital, and their experience, is that they feel stigmatised, and this … leads to premature discharge. And poor satisfaction amongst the drug‐using, treatment‐seeking cohort. So it's going to increase patient outcomes. It will increase nurses' ability to communicate effectively and confidently with drug using patients, because they'll be more understanding. And hopefully it will diminish some of the fear. There's a lot of fear because of the unknown. That unknown, hopefully will be broken down a little bit by having someone who is actively using some kind of drug… So it'll diminish things like stigma and stereotypes. (Nurse 2)

Consumers are raising it as an issue [health professional's stigma] so we need to look at that and address it. So I would hope that [co‐designed AOD education] would have a flow‐on effect. I just hope it'll make people [nurses] happier in their jobs too. If the patients are happy … the staff aren't stressed out. (Person with lived experience 3)

One of the participants with lived experience also commented that the value of co‐designing the education goes beyond reducing stigmatisation and improving AOD clinical care, noting that the co‐design process also helped them to give back to the community, which improved their self‐perception and sense of purpose in the world:

I used to have dreadful self‐esteem and have been trying to fix myself from, I think age 14… It's not until a couple of years ago, maybe two/three years ago that things finally have come together [speaking about starting to get involved in groups that advocate for people who use drugs], and finally I like myself and can speak well about myself. It still feels occasionally really awkward to say positive things about myself and to talk about myself as if I'm someone who can have a positive effect on the world. And it's like, I die even saying that but part of the expertise … I really do know that topic area. (Person with lived experience 1)

Findings from the value of the co‐design outcome theme indicate that participants perceived the potential value of a meaningful co‐design process for AOD education, which may provide a more accurate and humanistic portrayal of substance dependence and lead to reduced stigma and better clinical outcomes and opportunities for people who use substances.

## 5 Discussion

This qualitative study investigated the process of people with lived experience, clinicians and academics co‐designing an AOD education program for healthcare clinicians, with a specific focus on the barriers, enablers and value of the co‐design processes. The findings indicate that trusting and equitable relationships can be built in co‐design teams, and this is supported by participants' efforts towards collaboration, as well as by skilled facilitation and adequate resourcing. However, it took time to build trust, and nurses needed to be mindful not to be overbearing or use stigmatising language. The emphasis on equitable relationships has been highlighted as a factor in supporting marginalised people to engage in co‐design processes (Brand et al. 2021; Molloy et al. 2024; Mullins et al. 2021; River et al. 2023) and mirrors co‐design best practice principles (Clayson, Webb, and Cox 2018; Mckercher 2020).

Our findings indicate that clinicians can use stigmatising and language, and dominate the co‐design, which can be a barrier to developing trusting relationships. Stigma, language, and the power of clinicians have been noted previously as a barrier to co‐design (Bellingham et al. 2023; Roper, Grey, and Cadogan 2018). However, findings from our study indicate that it may be beneficial for participants to explicitly discuss power dynamics and stigma and agree on language use at the commencement of a project. The need for dialogue about power and language (Roper, Grey, and Cadogan 2018) and the option to develop an agreed project lexicon have been noted previously (Bellingham et al. 2023). The dialogue around power and acceptable language might be supported by power mapping processes outlined by Roper, Grey, and Cadogan (2018), which is a systematic approach to identifying, discussing, and attending to power differentials in co‐design or co‐production processes. Language guidelines such as those proposed in the Network of Alcohol and Other Drugs Agencies (NADA 2019) ‘Language Matters’ guide may support teams in determining acceptable, non‐stigmatising language about substance use.

Despite language and power dynamics challenges, participants in this study indicated that the co‐design process was supported by skilled facilitation, which was important for ensuring access, establishing a welcoming atmosphere and involving all members in the dialogue. As Mckercher (2020) stated, hospitality is vital to co‐design, making participants less anxious and more connected. Skilled facilitation may also support relational resilience in co‐design processes. However, our findings highlight the need for facilitators to maintain a careful balance between promoting access and ensuring team comfort. For example, promoting access by enabling members to bring dogs might inadvertently limit access for other participants who are uncomfortable with animals in the room. Additionally, the findings point to the need for facilitators to support, but not overly influence, the dialogue (Mckercher 2020). A trained facilitator, who is not a key decision‐maker, might best support this process.

Furthermore, our findings indicate that people with lived experience can adopt informal leadership roles that facilitate the involvement of lived experience members in the co‐design process. It has been noted previously that more egalitarian team structures can promote lived experience leadership, humanise co‐design teams, and promote relational resilience (River et al. 2023). Additionally, more formalised lived experience leadership or co‐leadership in co‐design processes can be particularly beneficial for attending to power imbalances, supporting facilitation, and embodying collaboration (Bellingham et al. 2021). Relational resilience refers to mutually empowering dynamics that support collective resilience in teams (Jordan 2004), which has been found to be important for supporting people with lived experience to express perspectives and navigate power dynamics (River et al. 2023). Co‐design may further benefit from a more formalised co‐facilitation approach, where people with lived experience are employed to facilitate co‐design teams in partnership (Bellingham et al. 2021).

Additionally, this study shows that having equal numbers of lived experience members in the co‐design team and creating an environment where diverse perspectives can be voiced and valued is important for collaborative processes. It has been proposed previously that equal numbers of people with lived experience in the co‐design process can reduce the impact of traumatising historical power differentials and support equitable engagement (Daya 2020; River et al. 2023; Roper, Grey, and Cadogan 2018). Therefore, in co‐design, lived‐experience team members are given more space and influence to ensure that power is distributed more evenly, and perspectives are acted upon (Daya 2020; Roper, Grey, and Cadogan 2018). However, a clear decision‐making process that ensures that people with lived experience have equitable power would be required, in addition to equal numbers, to ensure that lived experience involvement is not reduced to that of tokenism (i.e., views are heard but not acted upon) (River et al. 2023). River et al. (2023) also suggest that historical power imbalances in co‐design can be challenging for people with lived experience to navigate, and peer‐led mentoring and co‐learning spaces may be supportive.

This study highlights that a lack of resources could potentially disrupt the co‐design process. The need for adequate resourcing of co‐design—to ensure that people with lived experience are not disadvantaged in teams due to time limitations—has been noted previously (River et al. 2023). Although funding bodies are increasingly pushing for collaboration and co‐design for education, research, and service design and delivery, research indicates that projects are often hampered by inadequate funding and time (Jagtap 2022). This can lead to people with lived experience having less power and influence over the co‐design process (Fraser‐Barbour et al. 2023) and can hamper the equitable and continuous involvement of people with lived experience (Mckercher 2020; NSW Council of Social Services 2017).

Despite challenges related to the co‐design process, this study found that members valued the outcomes, which in this study was the development of AOD education that incorporated lived experience and clinical perspectives. Co‐designed education arguably has the potential to tackle systemic injustices, including stigmatising and discriminating attitudes against people who use substances, and could potentially contribute to improved healthcare for people who use substances. The value of co‐design projects for addressing social justice has been commented on in previous work (Mckercher 2020). However, our findings indicate that for the benefits of co‐designed education to be fully realised, people with lived experience perceive that co‐designed content needs to be co‐taught. The value of co‐designed and co‐taught AOD education, such as decreasing students' stigma and realising the importance of collaboration with people who use substances, has been reported in the context of undergraduate nursing education (Goodhew et al. 2023).

The value of the co‐design process may also extend beyond clinical practice, as findings indicate that for people with lived experience, it can also instil a sense of contributing to the community, enhance feelings of self‐efficacy, and lead to exciting work opportunities. A participatory action research project of the establishment of a consumer action group in a supervised injecting facility yielded similar findings (Goodhew 2019), with participants reporting that the opportunity to help their community increased their social capital, improved relationships, and provided new work opportunities, such as disseminating research and speaking at academic conferences.

## 6 Conclusions

This study investigated the experience of co‐designing AOD education, including barriers, facilitators, and the perceived value of the co‐design process. The findings indicate that the co‐design process is enhanced by skilled facilitation, fostering trusting and equitable relationships among team members. However, challenging interpersonal dynamics, inadequate resources, or overly directive facilitation are potential barriers to effective co‐design. Future recommendations include co‐facilitation of co‐design with lived experience partners. Authentic co‐design has the potential to transform AOD education, ensuring that it centres the needs of people with lived experience and requires adequate resourcing and time to develop equal and respectful relationships for this vision to be realised.

## 7 Relevance to Clinical Practice

The findings of this study provide guidance for optimising co‐design in clinical settings, including the importance of early conversations about power and language, skilled facilitation, and sufficient resource allocation. Clinicians should also consider co‐facilitating co‐design with lived experience partners to promote participation. The perceived value of co‐designed education lies in its potential to help clinicians see substance use through a more humanistic lens, reduce stigma, and improve treatment outcomes. This may lead people who use substances to be more willing to collaborate with clinicians in treatment and care.

## Author Contributions

Mark Goodhew and Jo River were involved in the study's conception and design, analysis and interpretation of the results, and drafting and approving the final manuscript. Sonia Matiuk and Carmen Axisa were involved in data collection, analysis and interpretation of the results, and drafting and approving the final manuscript. Chris Gough is a peer worker and lived experience advocate. He provided feedback on the research methodology and was involved in the writing of the manuscript.

## Disclosure

We declare we received no financial support or have relationships with any company that may pose a conflict of interest.

## Ethics Statement

This study has ethics approval through UTS, Sydney Human Ethics Committee Ref no. ETH21‐6148.

## Conflicts of Interest

The authors declare no conflicts of interest.

## Data Availability

Research data are not shared.

## References

[inm70009-bib-0001] Arblaster, K. , L. Mackenzie , N. Buus , et al. 2023. “Co‐Design and Evaluation of a Multidisciplinary Teaching Resource on Mental Health Recovery Involving People With Lived Experience.” Australian Occupational Therapy Journal 70: 354–365. 36704991 10.1111/1440-1630.12859

[inm70009-bib-0002] Australian Institute of Health and Welfare (AIHW) . 2022a. “Factsheet – Alcohol.” https://www.aihw.gov.au/getmedia/ab53a3c9‐90b6‐4f49‐8c58‐52828c12caef/PHE‐221‐Factsheets‐Alcohol.pdf.aspx.

[inm70009-bib-0003] Australian Institute of Health and Welfare (AIHW) . 2022b. “Illicit Drug Use.” https://www.aihw.gov.au/reports/illicit‐use‐of‐drugs/illicit‐drug‐use.

[inm70009-bib-0004] Barahmand, U. , A. Khazaee , and G. S. Hashjin . 2016. “Emotion Dysregulation Mediates Between Childhood Emotional Abuse and Motives for Substance Use.” Archives of Psychiatric Nursing 30: 653–659. 27888955 10.1016/j.apnu.2016.02.007

[inm70009-bib-0005] Bellingham, B. , B. Foxlwin , G. Rose , and J. River . 2023. “Co‐Production Kickstarter, Sydney, Community Mental Health Drug and Alcohol Research Network.”

[inm70009-bib-0006] Bellingham, B. , H. Kemp , K. Boydell , S. Isobel , K. Gill , and J. River . 2021. “Towards Epistemic Justice Doing: Examining the Experiences and Shifts in Knowledge of Lived Experience Researchers Over the Course of a Mental Health Research Training Programme.” International Journal of Mental Health Nursing 30: 1588–1598. 34263518 10.1111/inm.12910

[inm70009-bib-0007] Brand, G. , C. Sheers , S. Wise , et al. 2021. “A Research Approach for Co‐Designing Education With Healthcare Consumers.” Medical Education 55: 574–581. 33155301 10.1111/medu.14411

[inm70009-bib-0008] Braun, V. , and V. Clarke . 2022. Thematic Analysis: A Practical Guide. SAGE.

[inm70009-bib-0009] Brett, J. , S. Staniszewska , C. Mockford , et al. 2012. “Mapping the Impact of Patient and Public Involvement on Health and Social Care Research: A Systematic Review.” Health Expectations 17: 637–650. 22809132 10.1111/j.1369-7625.2012.00795.x PMC5060910

[inm70009-bib-0010] Campbell, P. , and D. Rose . 2010. “Action for Change in the UK: Thirty Years of the User/Survivor Movement.” In The Sage Handbook of Mental Health and Illness, edited by A. Rogers and B. Pescocolido . Sage.

[inm70009-bib-0011] Clayson, A. , L. Webb , and N. Cox . 2018. “When Two Worlds Collide: Critical Reflection on Co‐Production.” Drugs and Alcohol Today 18: 51–60.

[inm70009-bib-0012] Compton, P. , and S. Blacher . 2020. “Nursing Education in the Midst of the Opioid Crisis.” Pain Management Nursing 21: 35–42. 31358464 10.1016/j.pmn.2019.06.006

[inm70009-bib-0013] Copeland, D. 2020. “Drug‐Seeking: A Literature Review (And an Exemplar of Stigmatization in Nursing).” Nursing Inquiry 27: e12329. 31765066 10.1111/nin.12329

[inm70009-bib-0014] Daya, I. 2020. “The Participation Ladder: A Consumer/Survivor Lens.” Accessed October 21, 2024. https://www.indigodaya.com/wpcf7_captcha/2020/10/Participation‐ladder_consumer_survivor‐lens‐2.pdf.

[inm70009-bib-0015] Ford, R. 2011. “Interpersonal Challenges as a Constraint on Care: The Experience of Nurses' Care of Patients Who Use Illicit Drugs.” Contemporary Nurse 37: 241–252. 21692595 10.5172/conu.2011.37.2.241

[inm70009-bib-0016] Fraser‐Barbour, E. , S. Robinson , S. Gendera , et al. 2023. “Shifting Power to People With Disability in Co‐Designed Research.” Disability & Society 40: 1–22.

[inm70009-bib-0017] Goodhew, M. 2019. “Enhancing Consumer Participation in a Medically Supervised Injecting Centre Through Participatory Action Research.” Doctor of Philosophy Thesis, UTS, Sydney.

[inm70009-bib-0018] Goodhew, M. , J. River , Y. Samuel , et al. 2023. “Learning That Cannot Come From a Book: An Evaluation of an Undergraduate Alcohol and Other Drugs Subject Co‐Produced With Experts by Experience.” International Journal of Mental Health Nursing 32: 446–457. 36478635 10.1111/inm.13098

[inm70009-bib-0019] Goodhew, M. , Y. Samuel , C. Gilford , and J. River . 2021. “Co‐Producing a Drug and Alcohol Subject With Experts by Experience.” Australian Journal of Clinical Education 8: 1.

[inm70009-bib-0020] Goodhew, M. , J. Stein‐Parbury , and A. Dawson . 2018. “Consumer Participation in Drug Treatment: A Systematic Review.” Drugs and Alcohol Today 19: 97–112.

[inm70009-bib-0021] Happell, B. , C. Platania‐Phung , B. Scholz , et al. 2019. “Changing Attitudes: The Impact of Expert by Experience Involvement in Mental Health Nursing Education: An International Survey Study.” International Journal of Mental Health Nursing 28: 480–491. 30390371 10.1111/inm.12551

[inm70009-bib-0022] Happell, B. , S. Waks , J. Bocking , et al. 2019. “‘There's More to a Person Than What's in Front of You’: Nursing Students' Experiences of Consumer Taught Mental Health Education.” International Journal of Mental Health Nursing 28: 950–959. 30953420 10.1111/inm.12596

[inm70009-bib-0023] Horgan, A. , F. Manning , J. Bocking , et al. 2018. “‘To be treated as a human’: Using Co‐Production to Explore Experts by Experience Involvement in Mental Health Nursing Education—The COMMUNE Project.” International Journal of Mental Health Nursing 27: 1282–1291. 29377483 10.1111/inm.12435

[inm70009-bib-0024] Horner, G. , J. Daddona , D. J. Burke , J. Cullinane , M. Skeer , and A. G. Wurcel . 2019. “‘You're Kind of at War With Yourself as a Nurse’: Perspectives of Inpatient Nurses on Treating People Who Present With a Comorbid Opioid Use Disorder.” PLoS One 14: e0224335. 31648259 10.1371/journal.pone.0224335 PMC6812769

[inm70009-bib-0025] Jagtap, S. 2022. “Codesign in Resource‐Limited Societies: Theoretical Perspectives, Inputs, Outputs and Influencing Factors.” Research in Engineering Design 33: 191–211.

[inm70009-bib-0026] Jones, N. 2022. “Lived Experience Leadership in Peer Support Research as the New Normal.” Psychiatric Services 73: 125. 35100862 10.1176/appi.ps.73201

[inm70009-bib-0027] Jordan, J. V. 2004. “Relational Resilience.” In The Complexity of Connection: Writings From the Stone Center's Jean Baker Miller Training Institue, edited by J. Jordan , M. Walker , and L. M. Harting . Guilford Press.

[inm70009-bib-0028] Mckercher, K. A. 2020. Beyond Sticky Notes Co‐Design for Real: Mindsets, Methods and Movements. Beyond Sticky Notes.

[inm70009-bib-0029] Moll, S. , M. Wyndham‐West , G. Mulvale , et al. 2020. “Are You Really Doing ‘Codesign’? Critical Reflections When Working With Vulnerable Populations.” BMJ Open 10: e038339. 10.1136/bmjopen-2020-038339 PMC7640510 33148733

[inm70009-bib-0030] Molloy, R. , A. Hansen , E. Robinson , P. D'astoli , T. Wood , and N. Buus . 2024. “Stakeholder Perspectives on Co‐Designing a Post‐Registration Mental Health Nursing Curriculum: A Case Study.” Journal of Psychiatric and Mental Health Nursing 31: 303–312. 37822206 10.1111/jpm.12988

[inm70009-bib-0031] Mullins, R. M. , B. E. Kelly , P. Chiappalone , and V. J. Lewis . 2021. “‘No‐One Has Listened to Anything I've Got to Say Before’: Co‐Design With People Who Are Sleeping Rough.” Health Expectations 24: 930–939. 33756006 10.1111/hex.13235 PMC8235881

[inm70009-bib-0032] NADA . 2019. “Language Matters.” Accessed July 8, 2021. https://nada.org.au/wp‐content/uploads/2021/01/language_matters_‐_online_‐_final.pdf.

[inm70009-bib-0033] NADA . 2022. “Trauma‐Informed Practices for Responding to Difficult Situations.” Accessed November 26, 2024. https://nada.org.au/wp‐content/uploads/2022/12/Trauma‐informed‐practices.pdf.

[inm70009-bib-0034] National Health and Medical Research Council . 2018. “Consumer and Community Engagement.” Accessed December 27, 2023. https://www.nhmrc.gov.au/about‐us/consumer‐and‐community‐involvement/consumer‐and‐community‐engagement.

[inm70009-bib-0035] Neville, K. , and N. Roan . 2014. “Challenges in Nursing Practice: Nurses' Perceptions in Caring for Hospitalized Medical‐Surgical Patients With Substance Abuse/Dependence.” Journal of Nursing Administration 44: 339–346. 24835142 10.1097/NNA.0000000000000079

[inm70009-bib-0036] NSW Council of Social Services . 2017. “Principles of Co‐Design.” Accessed Febuary 19, 2024. https://www.ncoss.org.au/wp‐content/uploads/2017/06/Codesign‐principles.pdf.

[inm70009-bib-0037] Patterson, S. , J. Trite , and T. Weaver . 2014. “Activity and Views of Service Users Involved in Mental Health Research: UK Survey.” British Journal of Psychiatry 205: 68–75. 10.1192/bjp.bp.113.128637 24723628

[inm70009-bib-0038] Ritchie, H. 2019. “Drug Use.” Accessed November 7, 2021. https://ourworldindata.org/drug‐use.

[inm70009-bib-0039] River, J. , B. Bellingham , S. Isobel , et al. 2023. “Raising the Bar: A Qualitative Study of a Co‐Produced Model for Promoting Research Partnerships in Mental Health.” International Journal of Qualitative Methods 22: 16094069231213268.

[inm70009-bib-0040] Roper, C. , F. Grey , and E. Cadogan . 2018. “Co‐Production: Putting Principles Into Practice in Mental Health Contexts.” Melbourne University. Accessed December 7, 2021. Coproduction_putting‐principles‐into‐practice.pdf.

[inm70009-bib-0041] Roussy, V. , N. Thomacos , A. Rudd , and B. Crockett . 2015. “Enhancing Health‐Care Workers' Understanding and Thinking About People Living With Co‐Occurring Mental Health and Substance Use Issues Through Consumer‐Led Training.” Health Expectations 18: 1567–1581. 24118841 10.1111/hex.12146 PMC5060869

[inm70009-bib-0042] Sandelowski, M. 2010. “What's in a Name? Qualitative Description Revisited.” Research in Nursing & Health 33: 77–84. 20014004 10.1002/nur.20362

[inm70009-bib-0043] Simon, R. , R. Snow , and S. Wakeman . 2020. “Understanding Why Patients With Substance Use Disorders Leave the Hospital Against Medical Advice: A Qualitative Study.” Substance Abuse 41: 519–525. 31638862 10.1080/08897077.2019.1671942

[inm70009-bib-0044] Tierney, M. , D. S. Finnell , M. Naegle , A. M. Mitchell , and E. M. Pace . 2020. “The Future of Nursing: Accelerating Gains Made to Address the Continuum of Substance Use.” Archives of Psychiatric Nursing 34: 297–303. 33032749 10.1016/j.apnu.2020.07.010

[inm70009-bib-0045] UNODC . 2021. “World Drug Report.” Accessed November 7, 2021. https://www.unodc.org/unodc/en/data‐and‐analysis/wdr2021.html.

[inm70009-bib-0046] Valenti, M. , and K. Allred . 2020. “Using Peer Recovery Specialists to Educate Nursing Students.” Nurse Educator 45: 179–180. 31584480 10.1097/NNE.0000000000000738

[inm70009-bib-0047] van Boekel, L. C. , E. P. Brouwers , J. van Weeghel , and H. F. Garretsen . 2013. “Stigma Among Health Professionals Towards Patients With Substance Use Disorders and Its Consequences for Healthcare Delivery: Systematic Review.” Drug and Alcohol Dependence 131: 23–35. 23490450 10.1016/j.drugalcdep.2013.02.018

